# Permanent His-bundle Pacing in Pediatrics and Congenital Heart Disease

**DOI:** 10.19102/icrm.2020.110205

**Published:** 2020-02-15

**Authors:** Shannon Lyon, Gopi Dandamudi, Adam C. Kean

**Affiliations:** ^1^Department of Pediatrics, Division of Pediatric Cardiology, Indiana University School of Medicine, Indianapolis, IN, USA; ^2^Cardiovascular Service Line, CHI Franciscan, Tacoma, WA, USA; ^3^Pediatric Electrophysiology, Division of Pediatric Cardiology, Department of Pediatrics, Indiana University School of Medicine, Indianapolis, IN, USA

**Keywords:** Adult congenital heart disease, congenital heart disease, His-bundle pacing, pediatric cardiology

## Abstract

Permanent His-bundle pacing has been gaining popularity in the adult population requiring cardiac resynchronization therapy. Initial procedural challenges are being overcome, and this method of pacing has been shown to improve left ventricular function and heart failure symptoms secondary to ventricular dyssynchrony. Though the etiologies of ventricular dyssynchrony may differ in children and those with congenital heart disease than in adults with structurally normal hearts, His-bundle pacing may also be a preferred option in these groups to restore more physiologic electric conduction and improve ventricular function. We present a review of the current literature and suggested directions involving deploying permanent His-bundle pacing in the pediatric and congenital heart disease population.

## Introduction

The typical electrical activation of the heart propagates as an electrical impulse that travels from the sinus node to the atrioventricular (AV) node, through the His–Purkinje system, and to the ventricular myocardium. Conduction through the His–Purkinje system leads to a narrow QRS complex on the 12-lead electrocardiogram (ECG), representing synchronous left ventricular (LV) and right ventricular (RV) activation. In the setting of AV block, pacing of the ventricle (most commonly the RV) provides for an adequate ventricular heart rate. Chronic RV apical pacing leads to altered cardiac activation and mechanics with inherent left bundle branch (LBB) and ventricular dyssynchrony, resulting in reduced systolic function over time due to myocellular remodeling.^1–5^ The RV septum and outflow tract have been explored as alternative pacing sites and seem to improve ejection fractions in adults as compared with RV apical pacing, although randomized trials have not shown any differences.^6–9^ With the advent of biventricular pacemakers, the resynchronization of ventricular activation has led to clear morbidity and mortality benefits in the adult population. While effective in narrowing the QRS complex by creating a fused ventricular depolarization waveform, biventricular pacing is still unable to take advantage of the native His–Purkinje system and advantageous ventricular myocyte orientation for optimal electrical signal transduction. Permanent His-bundle pacing is an alternative method that may better restore physiologic ventricular activation.

## Selection

### Anatomy

In the structurally normal heart, the sinus node is located in the high right atrium at the superior vena cava–right atrial junction and initiates electrical excitation of the myocardium. The electric impulse takes an inferior and leftward course through the right atrium until it reaches the AV node. The AV node is an atrial structure situated between the AV valves posterior to the aortic valve at the apex of the triangle of Koch (which is delineated by the coronary sinus orifice, the tendon of Todaro, and the septal leaflet of the tricuspid valve). The AV node exhibits decremental conduction to the bundle of His that penetrates the AV plate, allowing for passage of the electric current to the ventricles. The electricity then travels through the bundle of His, in the membranous ventricular septum surrounded by insulating connective tissue. The bundle of His, though located in the membranous septum, is covered with myocardial fibers that travel through the membranous and muscular septum.^10^ This structure exits on the ventricular crest and travels to both bundle branches to deliver the electrical impulse to the LV and RV.^11^

### Epicardial versus transvenous pacing leads

**Figure 1:**
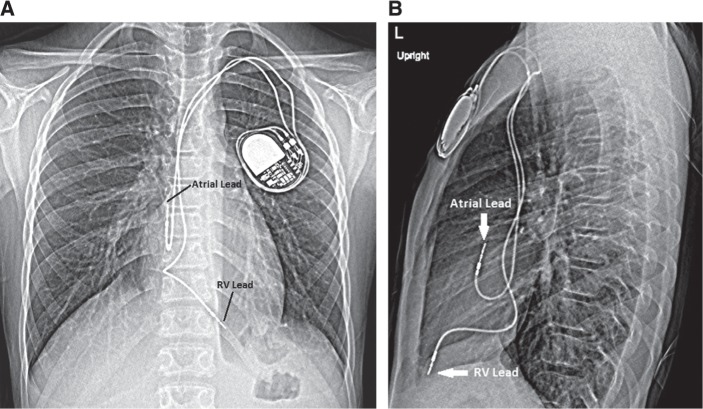
**A**: Anteroposterior chest radiograph showing lead placement in a dual-chamber transvenous pacing system in a structurally normal heart. **B**: Lateral chest radiograph showing lead placement in a dual-chamber transvenous pacing system in a structurally normal heart.

**Figure 2:**
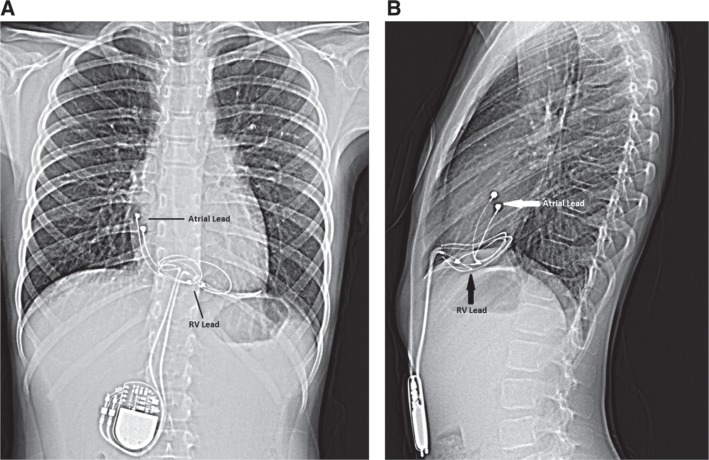
**A**: Anteroposterior chest radiograph showing lead placement of a bipolar dual-chamber epicardial pacing system in a structurally normal heart. **B**: Lateral chest radiograph showing lead placement of a bipolar dual-chamber epicardial pacing system in a structurally normal heart.

Pacemaker leads can be placed via a transvenous **(Figures 1A and 1B)** or epicardial **(Figures 2A and 2B)** route, which is largely determined by patient size and the presence of congenital heart disease (CHD). Adults with normal cardiac anatomy can typically accommodate a transvenous pacing system. Infants and young children most commonly benefit from epicardial pacemaker leads as the innominate vein size limits the ability to accommodate a transvenous lead without increased risk of obstruction.^12^ Heightened incidence rates of venous occlusion and thrombosis and the need for reintervention are challenges seen when implanting transvenous pacing systems in young children, especially those weighing less than 10 kg.^12,13^ Moreover, a small subxiphoid approach is often sufficient for a dual-chamber epicardial system in an infant or young child, making this technique very feasible. In those with CHD, intracardiac shunts limit the utility of transvenous pacing systems due to the increased risk of thromboembolism.^14,15^

In adults with ventricular dyssynchrony benefiting from biventricular pacing, the LV lead is traditionally placed transvenously via the coronary sinus.^16^ When this route is felt not to be available, surgical epicardial lead placement is also an option. Less commonly, a transvenous LV endocardial lead can be placed via a transseptal atrial puncture to allow access to the left atrium and thus the LV. This approach would require systemic anticoagulation.^17^ Small children with ventricular dyssynchrony are not able to accommodate a pacemaker lead in the coronary sinus or descending coronary veins and so commonly receive epicardial leads if biventricular pacing is desired.

### Cardiac resynchronization therapy in adults: biventricular and His-bundle pacing

Medical management for adults with chronic heart failure has improved morbidity and mortality rates; however, outcomes remain suboptimal.^18–20^ Cardiac resynchronization therapy (CRT) using a biventricular pacemaker increases LV ejection fraction, improves symptoms, quality of life, and decreases mortality for those with heart failure and ventricular dyssynchrony.^21–24^

His-bundle pacing is an alternative to biventricular pacing to restore ventricular synchrony. The goal of His-bundle pacing is to achieve physiologic ventricular activation where it otherwise cannot exist. This can be in cases of AV block (whether located at the AV node, proximal His bundle, or distal His bundle) requiring RV pacing or in patients with LBBB (whether surgical or myopathic). In the setting of permanent AV block, conventional endocardial pacing systems result in an obligate paced left LBBB morphology QRS, and these individuals are at a higher risk for ventricular dyssynchrony with decreased LV systolic function. As such, His-bundle pacing rather than RV myocardial pacing produces a physiologic narrow complex ventricular depolarization, which improves ejection fraction.

The feasibility of permanent His-bundle pacing was first demonstrated in canines by Karpawich et al. in 1992.^25^ Then, permanent His-bundle pacing was first demonstrated in humans in 2000 by Deshmukh et al.^26^ They described His-bundle pacing in 18 adults with chronic atrial fibrillation and dilated cardiomyopathy with a narrow QRS complex. Successful His-bundle pacing was accomplished in the majority of patients and improvement in LV function was seen in nine of those individuals who maintained His-bundle capture. Since this publication, multiple other studies have shown that His-bundle pacing leads to better ventricular function, improvement in New York Heart Association (NYHA) functional class, and a higher quality of life when compared with RV pacing.^27,28^

Patients who require a high percentage of ventricular pacing and thus who are at a high risk for developing pacemaker-induced cardiomyopathy may be individuals who will benefit the most from His-bundle pacing. Adult studies have demonstrated that these are patients with AV conduction disease and an otherwise narrow QRS complex, those with permanent atrial fibrillation, and those with failed biventricular CRT device implants.^29^ Similarly, individuals with profound LBBB and electrical and mechanical ventricular dyssynchrony are expected to regain a narrow complex QRS with His-bundle pacing and possible LV systolic function benefit. His-bundle pacing has been gaining acceptance in the adult population and may be a more ideal way to accomplish resynchronization in children who are too small to allow access to the coronary sinus for placement of an LV lead.

### Classification of His-bundle pacing

**Figure 3:**
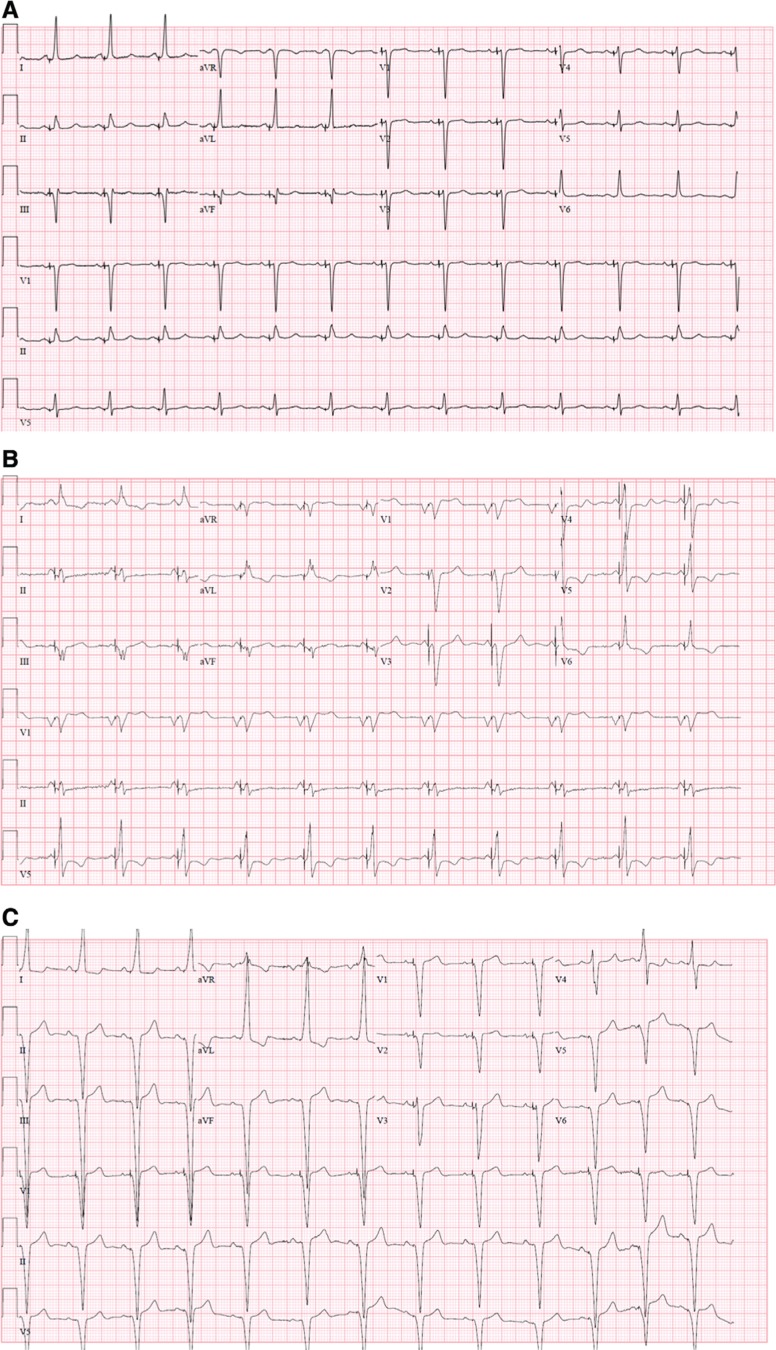
**A**: Selective His-bundle pacing in a patient with a structurally normal heart. **B**: Nonselective His-bundle pacing in a patient with LTGA. **C**: RV pacing in a structurally normal heart with a resultant LBBB pattern.

His-bundle pacing is accomplished either by direct stimulation of the His bundle (selective His-bundle pacing) or by stimulation of the His bundle and the surrounding ventricular tissue, defined as nonselective His-bundle pacing. There are published criteria to differentiate the two types of His-bundle pacing; essentially, selective His-bundle pacing results in His-bundle capture at low outputs and does not stimulate the ventricular myocardium as the His bundle is in direct contact with only the membranous septum, not the myocardium.^28,29^ Meanwhile, nonselective His-bundle pacing, may require higher outputs to stimulate the His bundle and involves ventricular capture due to placement of the lead closer to the ventricular myocardium.^28^ Both methods of His-bundle pacing should result in a narrower QRS complex as compared with isolated ventricular myocardial pacing **(Figures 3A–3C)**.

### Procedure

At present, the SelectSecure MRI SureScan 3830 transvenous lead (Medtronic, Minneapolis, MN, USA) is the only lead approved by the Food and Drug Administration for His-bundle pacing. This is a bipolar, nonretractable screw-in lead delivered via preformed catheters. An access sheath (7-French or larger) may be placed first to preserve vascular access following catheter removal in the event that final lead testing parameters are suboptimal, although this step is optional. The C315 His catheter (Medtronic, Minneapolis, MN, USA) is used to guide the transvenous lead into the correct position at the His bundle. The pacing lead is turned clockwise four to five times to affix the His-bundle helix. Sensing characteristics are tested and, if the transvenous lead is deemed to be in the appropriate position at the His bundle, a high-frequency His potential can be noted prior to the current of injury. When pacing is activated, a narrow, native QRS complex should be noted, indicating ideal placement of the His-bundle lead. An LBBB pacing morphology would indicate RV capture or right bundle branch capture. A fusion complex indicates nonselective His-bundle pacing, involving fused RV and His-bundle activation. Some providers have recently achieved LBB capture for resynchronization, which would result in a right bundle branch morphology.^30^

**Figure 4:**
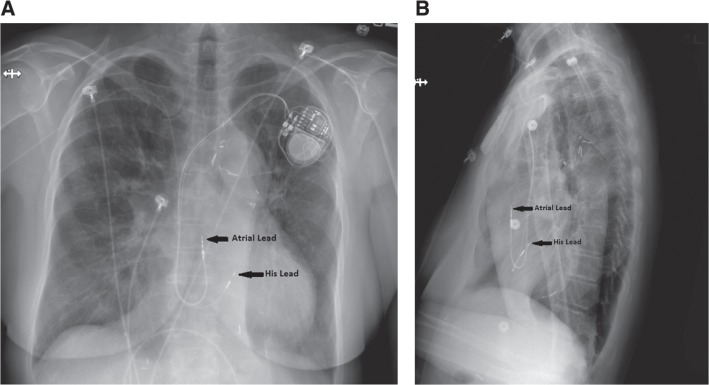
**A**: Anteroposterior chest radiograph showing lead placement in a His-bundle pacing system in a patient with a structurally normal heart. **B**: Lateral chest radiograph showing lead placement in a His-bundle pacing system in a patient with a structurally normal heart.

Once the His-bundle lead is in place, additional leads may be placed in a conventional fashion. For those who could benefit from resynchronization by way of His-bundle pacing, our preferred method for lead placement is to place an additional RV lead, particularly in the event of elevated His-lead threshold values. Finally, a lead is positioned in the right atrial appendage. All lead characteristics are retested through the CRT device with the His-bundle lead connected to the “left ventricular” port. **Figures 4A and 4B** demonstrate His-bundle lead placement when compared with the traditional RV dual-chamber transvenous pacemaker lead placement shown in **Figures 1A and 1B**.

### Challenges of His-bundle pacing

There are inherent challenges in optimal lead placement in His-bundle pacing: as an example, the membranous septum is thin and multiple attempts to screw in a lead can potentially result in perforation, although no cases of such have definitively been reported in the literature. Precise lead placement is needed to pace the His bundle at low outputs as opposed to the potential of requiring higher outputs with nonselective His-bundle pacing.^28,29^ However, experience in lead placement in adults is increasing and has been demonstrated to be safe and effective, with a success rate of 80% reported in one study by Sharma et al.^4^ More work needs to be done, however, regarding utilizing this technology in the pediatric and CHD populations.

## Application in pediatrics and congenital heart disease

### Anatomy of the conduction system in congenital heart disease

The positions of the AV node and bundle of His in certain congenital heart lesions differ from those in the structurally normal heart due to alterations in the embryologic migration of the four specialized myocardial rings that form the progenitor cardiac conduction system. As an example, in the context of a double-inlet LV, the AV node and bundle of His are displaced rightward where the AV valve ring meets the crest of the ventricular septum. In cases of congenitally corrected transposition of the great arteries, the AV node is displaced superiorly and rightward of the pulmonary valve, while the bundle of His travels anterosuperiorly to the pulmonary infundibulum.^31^ Those with right atrial isomerism often have two distinct AV nodes (ie, two distinct physical structures, not merely dual AV node physiology) conducting electricity to separate the bundle of His tissues.^32^ In patients with AV septal defects, the AV node is displaced posteroinferiorly between the atria and outside the triangle of Koch.^33^ All of these anatomic variations must be taken into consideration and may add procedural complexity when attempting to place a His-bundle pacemaker lead in patients with these congenital heart defects.

### Pacemaker lead placement

Data regarding optimal pacemaker lead sites are varied in pediatric patients. Janousek et al. studied the use of different pacing sites in 178 children with AV block and structurally normal hearts and found that pacing the LV apex or the LV midlateral wall resulted in higher shortening fraction and ejection fraction than when using any RV pacing site.^8^ Karpawich et al. in 2015 assessed various pacing sites along the RV septum and apex in patients with and without CHD, finding that 38% of cases showed optimal contractility when paced at the RV midseptum; however, in this cohort, among patients with CHD, the optimal pacing site varied significantly.^34^ The difference in results between these studies is likely accounted for by the different populations (with versus without CHD) and the variation in pacing sites. The heterogeneity in pediatric patients with CHD likely necessitates an individualized approach to ventricular pacing sites.

Epicardial leads may be favored in those with CHD who have residual shunt lesions, as transvenous systems may increase the risk of paradoxical emboli.^14,15^ In those with altered systemic venous anatomy—for example, those palliated with a cavopulmonary connection Fontan procedure—transvenous pacing is not optimal in the absence of systemic anticoagulation. As previously discussed, small children typically require epicardial leads as their systemic veins are not large enough to accommodate a transvenous pacing system. In regard to CRT in the pediatric population, this is often achieved with epicardial lead placement.

### Cardiac resynchronization therapy in pediatrics

The indications and efficacy of CRT in pediatrics and those with CHD are yet to be fully characterized. These populations differ from adults potentially due to the longer overall duration of pacemaker dependence and difference in anatomic substrate in the case of CHD. A study conducted in 2005 by Dubin et al. retrospectively assessed biventricular pacing in patients younger than 21 years of age or who had CHD. The majority of patients in this study had reduced LV systolic function and CHD, which differs from the profile of the typical adult patient requiring CRT. It was determined that CRT was beneficial in improving ejection fraction and shortening QRS duration in these populations.^35^ A subsequent study conducted by Janousek et al. concluded that pacemaker-induced cardiomyopathy in the setting of CHD or pediatric status is the most significant indication for CRT therapy.^36^ This study found that, of those with CHD, individuals with a systemic LV had the most improvement in ejection fraction and the largest decrease in the LV end-diastolic dimension. The second significant predictor of CRT success was a higher grade of initial systemic AV valve regurgitation, which improved after CRT pacing. As such, while indications may differ in pediatrics and in those with CHD, there may indeed be a benefit to CRT in select groups. Regardless of the substrate, CRT is indicated in those who do have ventricular dyssynchrony leading to altered cardiac mechanics.

### His-bundle pacing in pediatrics and congenital heart disease

While His-bundle pacing is being used with increasing frequency in adult patients, experience with its application in pediatric patients and those with CHD has been more limited. In contrast to adults, pediatric patients face considerations such as a longer duration of pacing, anatomic size limitations that may impact the choice of pacemaker device, and ongoing somatic growth. This subset of patients who experience ventricular dyssynchrony from long-term pacing or who are too small to accommodate a coronary sinus lead for biventricular pacing may benefit from His-bundle pacing to restore ventricular synchrony. Data in this population, however, are currently lacking.

Children with CHD—another special population—are surviving longer into adulthood due to improved surgical and medical treatments but are also now experiencing secondary long-term complications in greater numbers, including heart failure and ventricular dyssynchrony.^37^ While adult criteria to initiate CRT can be extrapolated to pediatric and congenital heart populations in certain circumstances, this strategy has clear limitations in generalizing results to a different population. At present, many patients with CHD are treated with CRT to prevent complications associated with chronic RV pacing.^38^

**Figure 5:**
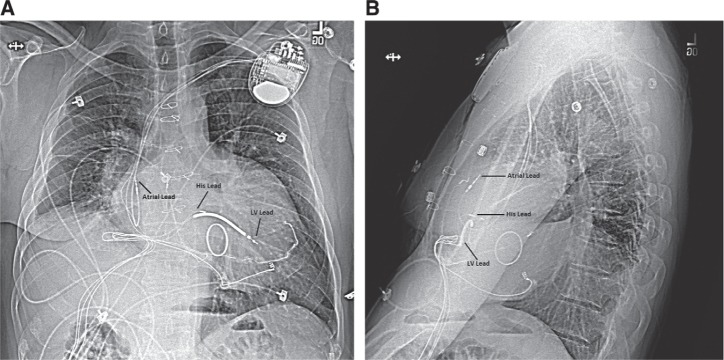
**A**: Anteroposterior chest radiograph showing lead placement in a His-bundle pacing system in a patient with LTGA. **B**: Lateral chest radiograph showing lead placement in a His-bundle pacing system in a patient with LTGA.

As noted previously, those with CHD also have special anatomic considerations. The AV node in these patients may be displaced in certain lesions, while transvenous access to the coronary sinus may not be possible for biventricular pacing. The successful implantation of a His-bundle pacemaker has been described before in an adult patient with L-transposition of the great arteries (LTGA) and complete AV block^39^ **(Figures 5A and 5B)**. This type of pacing was an attractive option, given the difficulty in cannulating the coronary sinus for CRT. Subsequent follow-up revealed no decrease in systemic ventricular systolic function despite chronic atrial fibrillation and 100% ventricular pacing (personal communication). Further studies are needed in those with CHD to better assess the feasibility and long-term outcomes for His-bundle pacing in this population.

## Conclusions

His-bundle pacing has been consistently gaining popularity in the adult population requiring CRT. Initial procedural challenges are being overcome and this method of pacing has been shown to improve LV function and heart failure symptoms secondary to ventricular dyssynchrony. Although the etiologies of ventricular dyssynchrony may differ between children and those with CHD and adults with structurally normal hearts, His-bundle pacing may be a preferred option in these populations to restore more physiologic electric conduction and improve ventricular function.

## References

[1] Sweeney MO, Hellkamp AS, Ellenbogen KA (2003). Adverse effect of ventricular pacing on heart failure and atrial fibrillation among patients with normal baseline QRS duration in a clinical trial of pacemaker therapy for sinus node dysfunction.. Circulation..

[2] Little WC, Reeves RC, Arciniegas J, Katholi RE, Rogers EW (1982). Mechanism of abnormal interventricular septal motion during delayed left ventricular activation.. Circulation..

[3] Shimony A (2012). Beneficial effects of RV non-apical vs apical pacing: a systematic review and meta-analysis or randomized-controlled trials.. Europace..

[4] Sharma PS, Ellenbogen KA, Trohman RG (2017). Permanent his bundle pacing: the past, present, and future.. J Cardiovasc Electrophysiol..

[5] Zhang XH, Chen H, Siu CW (2008). New-onset heart failure after permanent right ventricular apical pacing in patients with acquired high-grade atrioventricular block and normal left ventricular function.. J Cardiovasc Electrophysiol..

[6] Padeletti L, Lieberman R, Valsecchi S, Hettrick DA (2006). Physiologic pacing: new modalities and pacing sites.. Pacing Clin Electrophysiol..

[7] Kaye GC, Linker NJ, Marwick TH (2015). Effect of right ventricular pacing lead site on left ventricular function in patients with high-grade atrioventricular block: results of the Protect Pace study.. Eur Heart J..

[8] Janousek J, van Geldorp IE, Krupičková S (2013). Permanent cardiac pacing in children: choosing the optimal pacing site: a multicenter study.. Circulation..

[9] Raichlen JS, Campbell FW, Edie RN, Josephson ME, Harken AH (1984). The effect of the site of placement of temporary epicardial pacemakers on ventricular function in patients undergoing cardiac surgery.. Circulation..

[10] Kawashima T, Sasaki H (2005). A macroscopic anatomical investigation of atrioventricular bundle locational variation relative to the membranous part of the ventricular septum in elderly human hearts.. Surg Radiol Anat..

[11] Mulpuru SK, Cha YM, Asirvatham SJ (2016). Synchronous ventricular pacing with direct capture of the AV conduction system: functional anatomy, terminology, and challenges.. Heart Rhythm..

[12] Vos LM, Kammeraad JAE, Freund MW, Blank AC, Breur JMPJ (2017). Long-term outcome of transvenous pacemaker implantation in infants: a retrospective cohort study.. Europace..

[13] Chandler SF, Fynn-Thompson F, Mah DY (2017). Role of cardiac pacing in congenital complete heart block.. Expert Rev Cardiovasc Ther..

[14] Takeuchi D, Tomizawa Y (2013). Pacing device therapy in infants and children: a review.. J Artif Organs..

[15] McLeod CJ, Attenhofer Jost CH, Warnes CA (2010). Epicardial versus endocardial permanent pacing in adults with congenital heart disease.. J Interv Card Electrophysiol..

[16] Oddone D, Solari D, Nangah R (2017). Optimization of coronary sinus lead placement targeted to the longest right-to-left delay in patients undergoing cardiac resynchronization therapy: the Optimal Pacing SITE 2 (OPSITE 2) acute study and protocol.. Pacing Clin Electrophysiol..

[17] Garland V, Singh JP, Leclercq C (2019). Alternative left ventricular pacing approaches for an optimal cardiac resynchronization therapy.. Heart Rhythm..

[18] The CONSENSUS Trial Study Group. (1987). Effects of enalapril on mortality in severe congestive heart failure: results of the Cooperative North Scandinavian Enalapril Survival Study (CONSENSUS).. N Engl J Med..

[19] Bristow MR (2000). Beta-adrenergic receptor blockade in chronic heart failure.. Circulation..

[20] Pitt B, Zannad F, Remme WJ (1999). The effect of spironolactone on morbidity and mortality in patients with severe heart failure. Randomized Aldactone Evaluation Study Investigators.. N Engl J Med..

[21] Cleland JG, Daubert JC, Erdmann E (2005). The effect of cardiac resynchronization on morbidity and mortality in heart failure.. N Engl J Med..

[22] Cazeau S, Leclercq C, Lavergne T (2001). Effects of multisite biventricular pacing in patients with heart failure and intraventricular conduction delay.. N Engl J Med..

[23] Bristow MR, Saxon LA, Boehmer J (2004). Cardiac-resynchronization therapy with or without an implantable defibrillator in advanced chronic heart failure.. N Engl J Med..

[24] Daubert C, Gold MR, Abraham WT (2009). Prevention of disease progression by cardiac resynchronization therapy in patients with asymptomatic or mildly symptomatic left ventricular dysfunction: insights from the European cohort of REVERSE trial.. J Am Coll Cardiol..

[25] Karpawich PP, Gates J, Stokes KB (1992). Septal His Purkinje ventricular pacing in canines: a new endocardial electrode approach.. Pacing Clin Electophysiol..

[26] Deshmukh P, Casavant DA, Romanyshyn M, Anderson K (2000). Permanent direct His bundle pacing: a novel approach to cardiac pacing in patients with normal His-Purkinje activation.. Circulation..

[27] Occhetta E, Bortnik M, Magnani A (2006). Prevention of ventricular desynchronization by permanent para-Hisian pacing after atrioventricular node ablation in chronic atrial fibrillation: a crossover, blinded, randomized study versus apical right ventricular pacing.. J Am Coll Cardiol..

[28] Sharma PS, Dandamudi G, Naperkowski A (2015). Permanent His-bundle pacing is feasible, safe, and superior to RV pacing in routine clinical practice.. Heart Rhythm..

[29] Deshmukh PM, Romanyshyn M (2004). Direct His-bundle pacing: present and future.. Pacing Clin Electrophysiol..

[30] Hou X, Qian Z, Wang Y (2019 Jul 19). Feasibility and cardiac synchrony of permanent left bundle branch pacing through the interventricular septum.. Europace..

[31] Anderson RH, Becker AE, Arnold R, Wilkinson JL (1974). The conducting tissues in congenitally corrected transposition.. Circulation..

[32] Wu MH, Lin JL, Wang JK, Chiu IS, Young ML (1995). Electrophysiological properties of dual atrioventricular nodes in patients with right atrial isomerism.. Br Heart J..

[33] Thiene G, Wenink AC, Frescura C (1981). Surgical anatomy and pathology of the conduction tissues in atrioventricular defects.. J Thorac Cardiovasc Surg..

[34] Karpawich PP, Singh H, Zelin K (2015). Optimizing paced ventricular function in patients with and without repaired congenital heart disease by contractility-guided lead implant.. Pacing Clin Electrophysiol..

[35] Dubin AM, Janousek J, Rhee E (2005). Resynchronization therapy in pediatrics and congenital heart disease patients: an international multicenter study.. J Am Clin Cardiol..

[36] Janousek J, Gebauer RA, Abdul-Khaliq H (2009). Cardiac resynchronization therapy in paediatric and congenital heart disease: differential effects in various anatomical and functional substrates.. Heart..

[37] Diller GP, Kempny A, Alonso-Gonzalez R (2015). Survival prospects and circumstances of death in contemporary adult congenital heart disease patients under follow-up at a large tertiary centre.. Circulation..

[38] Flugge AK, Wasmer K, Orwat S (2018). Cardiac resynchronization therapy in congenital heart disease: results from the German National Register for Congenital Heart Defects.. Int J Cardiol..

[39] Kean AC, Kay WA, Patel JK, Miller JM, Dandamudi G (2017). Permanent nonselective His bundle pacing in an adult with L-TGA and complete AV block.. Pacing Clin Electrophysiol..

